# Supplementary material for: Comparison of time to death and its predictors among hospitalized children with and without severe acute malnutrition at Mulanje District Hospital, Southern Malawi
Source: PLoS One. 2025 Sep 30;20(9):e0333585. doi: 10.1371/journal.pone.0333585 (PMC12483230; doi:10.1371/journal.pone.0333585)
Supplement: Attachment — Submitted filename: Response to Reviewers.pdf [file pone.0333585.s001.pdf]

Kamuzu University of Health Sciences
Private Bag 360
Blantyre, Malawi

6th January, 2024

To: The Chief Editor
PLOS Publication
Reference: PONE-D-23-41003

Dear Sir/Madam,

RE-SUBMISSION OF MANUSCRIP FOR PUBLICATION

We would like to re-submit our manuscript for publication. We sincerely believe that this study was conducted in accordance with ethical requirements as recommended by the PLOS.

Since it was a retrospective cohort study, it was practically impossible to collect individual informed consent from individual participants or caregivers. However, the study protocol was ethically reviewed and approved by the Kamuzu University of Health Sciences before conducting the study. The study protocol was also presented at Mulanje District Hospital for ethical approved, and was approved by the Mulanje District Hospital Research Coordinating Committee. Besides this, this study was reviewed and approved by the research regulatory body in Malawi, the College of Medicine Research and Ethics Committee. We have uploaded all the approval letters for your review.

We are looking forward to hearing from you.

Yours Sincerely,

Authors

(Masuzgo Pajogo)
(Mirium Ndhlovu)
