# Supplementary material for: Comparison of time to death and its predictors among hospitalized children with and without severe acute malnutrition at Mulanje District Hospital, Southern Malawi
Source: PLoS One. 2025 Sep 30;20(9):e0333585. doi: 10.1371/journal.pone.0333585 (PMC12483230; doi:10.1371/journal.pone.0333585)
Supplement: Attachment — Submitted filename: Response to reviewers.docx [file pone.0333585.s002.docx]

**Point-by-point response file**

| **Reviewer 1** | | |
| --- | --- | --- |
| Comment | Response | Sub-section |
| In the Introduction, it does not come out clearly what the Authors set out to do in their study | The introduction has been revised and has clearly highlighted what authors set out to conduct | Introduction |
| Inclusion and exclusion criteria are also not clear to the reader. | The exclusion and inclusion criteria are revised. Ages of patients who were excluded is clearly indicated. Incomplete charts is no longer mentioned. | Inclusion and exclusion criteria |
| On sampling, it is not clear to me how the sampling was done once the Authors came out with a SAM group and a non-SAM group | The sampling process has been revised and it has clearly explained what authors did. | Sampling |
| In any case, it doesn't come out clearly in their title and introduction, that their study is about comparing predictors of mortality in children with SAM and those without SAM | The title has been changed to comparison of time to death and predictors of mortality between under-five children with severe acute malnutrition and without severe acute malnutrition hospitalized at Mulanje District Hospital in Southern Malawi; A retrospective cohort study. | Title |
| In Figure 3, Figure 4 and Table 5, the Authors now introduce a new concept, Survival function that wasn't anywhere in their 'not so clear' objectives. This is misplaced. | Figure 3, Figure 4, and Table 5 have been removed. | Results |
| In the results section, The Authors bring out the Mortality rate and time to death. They also bring about the predictors of mortality but present them as if they had set out to compare predictors of mortality in children with SAM and in children without SAM. if this was the intention, it should be clarified right from the title of the study to the introduction and methods. | The title and objectives have been revised so that reader should know that the study was aimed at comparing time to death and its predictors between SAM and non-SAM children | Title and objectives |
| The authors discuss the predictors of mortality from a point of why they are different between the two populations. I think they should be discussing why shock and not using Amoxicillin were predictive of mortality and also relating to other studies | The discussion has been revised. It is now discussing why the identified predictors were significant. It is also comparing the results from other similar studies. | Recruitment process |
| The Authors also dedicate three paragraphs in the discussion section to discussing what was not predictive of mortality in their study before finally discussing what was. This needs serious revision. The entire discussion section seems disconnected from the objectives of the study, if I got them well from the Abstract | This has been revised. The authors have focused on areas which were identified as significant predictors. | Discussion |
| Additionally, when discussing about shock, they focus on discussing shock in non-SAM children not children with SAM who are the major focus of the study. | This has been revised. The focus in now on children with SAM. | Discussion |
| The Authors pool all predictors of mortality (in SAM and non-SAM) children together, which creates the impression that all the variables given are predictors of mortality in children with SAM which is not true. | This has been revised. Only those variable that had p-value of less than 0.05 have been considered. We have also included variables which were identified as significant predictors in the previous | Discussion |
| **Reviewer 2** | | |
| 1. The paper could report descriptive statistics to compare if the average or median values of predictors and outcomes between the two groups are statistically different. This analysis would explain any significant differences between SAM and non-SAM cohorts. Strengthen its statistical validity. | The paper has included descriptive statistics to compare if the average or median values of predictors and outcomes between the two groups are statistically different | Results |
| 2. It needs further elaboration on SAM vs. non-SAM disparities in terms of their treatments. Understanding these distinctions is crucial for comprehending the observed outcomes. Highlighting the specific interventions, nutritional strategies, and medical protocols tailored for each group would enhance the paper's comprehensiveness. | In the revised paper, we have elaborated on SAM vs. non-SAM disparities in terms of their treatments | Introduction |
| 3. It's essential to address why this research is significant in the context of existing literature and how its findings can inform healthcare policies and clinical practices. Discussing potential interventions or guidelines stemming from the study's results would augment its impact | In the revised introduction, we have highlighted why this research was significant in the context of existing literature and how its findings informed healthcare policies and clinical practices | Introduction |
| **Academic editor** | | |
| Please ensure that the manuscript meet PLOS ONE’s style requirements | The linked were opened. The manuscript was revised based on required highlighted in the links. The revised manuscript meet PLOS ONE’s style requirements | The whole manuscript |
| PLOS journal require all data underlying the findings described in the manuscript be freely available to other researchers | In the new statement has been included, highlighting that data will be made freely available to other researchers | Data availability |
| If any Table files show item type figure/ other , please change type to Table | All table files which have the name of figures or others have been named as Tables | Portal |
| Please clarify the objectives and ensure that the design and analysis align with the study objectives. | The study objective has been clearly clarified. It now aligns with the design and analysis | Abstract and introduction |
| It is unclear why children without SAM were included. The mention of exposed and unexposed children based on SAM is confusing | The Non-SAM children were included so that they should be compared with SAM children in terms of time to death and predictors of mortality. | Abstract and introduction |
| Exposures should be the predictors of interest | All predictors of interest were considered as exposures | Methods and results |
| In the regression analysis, consider that some variables may be on the causal pathway and including them in the same model may attenuate the HR of distal variables. | All variables that could have existed in the causal pathway were completely removed to avoid attenuating the HR of distal variables. | Results |
