# Supplementary material for: Comparison of time to death and its predictors among hospitalized children with and without severe acute malnutrition at Mulanje District Hospital, Southern Malawi
Source: PLoS One. 2025 Sep 30;20(9):e0333585. doi: 10.1371/journal.pone.0333585 (PMC12483230; doi:10.1371/journal.pone.0333585)
Supplement: Attachment — Submitted filename: The response to reviewers.docx [file pone.0333585.s003.docx]

**Point-by-point response file**

| **Reviewer 2** | | |
| --- | --- | --- |
| Comment | Response | Sub-section |
| The current title is quite lengthy and specific. A more concise and focused title would enhance readability and better capture the essence of the study. | The title of the paper has been revised to: “Comparison of Time to Death and Its Predictors in Hospitalized Children with and without Severe Acute Malnutrition at Mulanje District Hospital, Southern Malawi.” This revision makes the title more concise and focused. | Title. |
| Inpatient Treatment Outcome: The statement regarding "one-third" is ambiguous. Is it supposed to be two-third? | In the revised version, it has been clearly emphasized that it was "two-thirds," not "one-third." | Results |
| The term "total follow-up time" needs clarification. It is important to specify whether this refers to the cumulative number of days across all children in the study or the duration each individual child was monitored. | The follow-up time has been revised to clearly indicate that it represents the cumulative number of days across all children in the study. | Results |
| Non-Significant Variables: The discussion primarily focuses on significant variables while excluding non-significant ones. It would be beneficial to discuss why certain variables, particularly those related to malaria, were not significant and how this might affect the overall findings. | In the revised version, several non-significant variables are discussed, including HIV, age, sex, and the use of NGT tubes. | Discussion |
| Discharge Proportions: Although the discharge proportions are similar between the two groups (SAM and non-SAM), the mortality rates differ significantly. It would be helpful to explore whether the outcomes for non-SAM children post-discharge were tracked and if these might influence the observed mortality differences. | This study did not track the outcomes of non-SAM children post-discharge. In the revised version, this has been addressed in both the discussion and the study limitations section. | Discussion and limitations. |
| Malaria as a Confounder: The higher mortality rate observed in non-SAM children may be influenced by severe malaria, which introduces a potential confounding factor. This should be explicitly acknowledged as a limitation of the study, with a discussion on how it impacts the comparability between groups. | The revised version explicitly states that severe malaria acted as a confounder, potentially leading to an overestimation of the mortality rate in non-SAM children compared to those with SAM. | Limitations. |
| Discussion: The discussion section could benefit from a more comprehensive exploration of the results. | This section has been revised to include a discussion of both significant and non-significant variables, making it more comprehensive than before. | Discussion |
