# Supplementary material for: Comparison of time to death and its predictors among hospitalized children with and without severe acute malnutrition at Mulanje District Hospital, Southern Malawi
Source: PLoS One. 2025 Sep 30;20(9):e0333585. doi: 10.1371/journal.pone.0333585 (PMC12483230; doi:10.1371/journal.pone.0333585)
Supplement: Attachment — Submitted filename: The_response_to_reviewers_auresp_3.docx [file pone.0333585.s004.docx]

**Point-by-point response file**

| **Reviewer 2** | | |
| --- | --- | --- |
| Comment | Response | Sub-section |
| It is necessary to address the potential for misinterpretation regarding the observed difference in time to death between children with Severe Acute Malnutrition (SAM) (5 days) and Non-SAM (1 day). To prevent the misconception that SAM is "less severe" or "less critical" than Non-SAM, the authors should explicitly emphasize that this disparity in time to death does not indicate a lower severity of SAM | In the revised manuscript, we have clearly emphasized that this discrepancy does not suggest that SAM is less critical. Rather, the observed difference likely reflects variations in underlying biological mechanisms, distinct causes of mortality, and differences in the medical interventions provided. We have also highlighted that the slower progression to death among children with SAM may be attributed to physiological adaptations to prolonged malnutrition. In contrast, non-SAM children often die more rapidly due to acute and highly fatal conditions that can lead to sudden systemic failure. | Discussion |
