# Supplementary material for: Comparison of time to death and its predictors among hospitalized children with and without severe acute malnutrition at Mulanje District Hospital, Southern Malawi
Source: PLoS One. 2025 Sep 30;20(9):e0333585. doi: 10.1371/journal.pone.0333585 (PMC12483230; doi:10.1371/journal.pone.0333585)
Supplement: Attachment — Submitted filename: Comments to the authors.docx [file pone.0333585.s005.docx]

**Comments to the authors**

In this study, the authors tried to address a critical gap in pediatric care in resource-limited settings. Its robust methodology and focus on understudied predictors are strengths.

The topic has the potential of contributing to the existing scientific knowledge on time to death among children with SAM and non-SAM.

I have the following major concerns, which the authors need to consider substantially.

**Abstract**

1. Overly dense; could benefit from brevity. The higher mortality in non-SAM children is not contextualized, which might confuse readers given SAM’s established risks.
2. Clarify why non-SAM mortality exceeds SAM despite SAM’s known severity.

**Introduction**

3. Transition from global to local context is abrupt. Limited data on Malawi’s SAM mortality post-2015.
4. Add recent Malawi-specific mortality statistics. Emphasize the clinical relevance of comparing SAM/non-SAM predictors.

**Methods**

5. Missing data handling and potential biases (e.g., selection bias due to excluded surgical cases) underdiscussed.
6. No mention of inter-rater reliability for data collectors.
7. Address how missing data were managed.
8. Justify exclusion of surgical patients more thoroughly.
9. Describe training for data collectors to ensure consistency.

**Results**

10. Generally the result presentation is not organized and needs major revision.
11. Extremely wide CI for non-SAM amoxicillin predictor (33.49; 4.47–250.7) suggests imprecision.
12. **Mortality rate discrepancies (non-SAM > SAM) are not statistically contextualized**.
13. Include visual aids (e.g., **survival curves**). Consider also description of the non-parametric use of the survival curves in the method section.
14. Discuss the clinical significance of large effect sizes (e.g., amoxicillin’s role in non-SAM).

**Discussion**

15. Under addresses why non-SAM mortality surpasses SAM despite SAM’s severity. Limited exploration of oxygen therapy’s risks (e.g., toxicity).
16. Expand on confounding factors (e.g., severe malaria’s role in non-SAM deaths). Discuss antibiotic resistance implications for amoxicillin recommendations.

**Conclusions and Recommendations**

17. Overlooks antibiotic stewardship concerns.
18. Recommendations lack specificity (e.g., *how* to optimize oxygen therapy).
19. Advocate for protocol revisions (e.g., timing of antibiotics, oxygen titration).
20. Highlight need for resource allocation (e.g., amoxicillin access).
