# Supplementary material for: Comparison of time to death and its predictors among hospitalized children with and without severe acute malnutrition at Mulanje District Hospital, Southern Malawi
Source: PLoS One. 2025 Sep 30;20(9):e0333585. doi: 10.1371/journal.pone.0333585 (PMC12483230; doi:10.1371/journal.pone.0333585)
Supplement: Attachment — Submitted filename: The response to reviewers(1).docx [file pone.0333585.s006.docx]

**Point-by-point response file**

| **No.** | **Reviewer Comment** | **Response** | **Section** |
| --- | --- | --- | --- |
| 1 | Overly dense; could benefit from brevity. The higher mortality in non-SAM children is not contextualized, which might confuse readers given SAM’s established risks. | The abstract has been revised for clarity and brevity. We have now contextualized the higher mortality in non-SAM children by highlighting relevant clinical factors. | Abstract |
| 2 | Clarify why non-SAM mortality exceeds SAM despite SAM’s known severity. | The revised abstract now explains that the higher mortality among non-SAM children may be attributed to more acute disease presentations and delayed interventions. | Abstract |
| 3 | Transition from global to local context is abrupt. Limited data on Malawi’s SAM mortality post-2015. | The introduction now includes more global context before transitioning to local data. Additional post-2015 Malawi-specific data has been added. | Introduction |
| 4 | Add recent Malawi-specific mortality statistics. Emphasize the clinical relevance of comparing SAM/non-SAM predictors. | Recent Malawi mortality data has been incorporated. The clinical relevance of comparing SAM and non-SAM predictors is now explicitly stated. | Introduction |
| 5 | Missing data handling and potential biases (e.g., selection bias due to excluded surgical cases) underdiscussed. | Missing data were handled using mean imputation, now clearly explained in the revised manuscript. Potential biases, including those from surgical case exclusion, are addressed in the limitations section. | Methods & Limitations |
| 6 | No mention of inter-rater reliability for data collectors. | Inter-rater reliability details have been added under the data quality control section. | Methods |
| 7 | Address how missing data were managed. | Missing data were managed using mean imputation. This is now clearly described in the methodology. | Methods |
| 8 | Justify exclusion of surgical patients more thoroughly. | The rationale for excluding surgical patients has now been clearly justified in the methods section. | Methods |
| 9 | Describe training for data collectors to ensure consistency. | Training procedures for data collectors are now fully described in the methodology. | Methods |
| 10 | Results presentation is disorganized and needs major revision. | The results section has undergone major revision and is now organized and clearly structured. | Results |
| 11 | Extremely wide CI for non-SAM amoxicillin predictor (33.49; 4.47–250.7) suggests imprecision. | Upon re-analysis, we identified a typographical error. The correct CI is (33.49; 4.47–45.07), which has been updated accordingly. | Results |
| 12 | Mortality rate discrepancies (non-SAM > SAM) are not statistically contextualized. | We have now provided appropriate statistical context for the observed mortality rate discrepancies. | Results |
| 13 | Include visual aids (e.g., survival curves). Consider also describing non-parametric use in methods. | Kaplan-Meier curves have been re-included as per this recommendation, though they were previously removed based on earlier feedback. Their use is now also described in the methods. | Methods & Results |
| 14 | Discuss the clinical significance of large effect sizes (e.g., amoxicillin’s role in non-SAM). | The clinical implications of large effect sizes, particularly for amoxicillin in non-SAM patients, are now discussed in detail. | Results |
| 15 | Under-addresses why non-SAM mortality surpasses SAM despite SAM’s severity. Limited exploration of oxygen therapy’s risks (e.g., toxicity). | We now fully address the unexpected higher mortality in non-SAM cases and explore potential risks of oxygen therapy, including toxicity. | Discussion |
| 16 | Expand on confounding factors (e.g., severe malaria’s role in non-SAM deaths). Discuss antibiotic resistance implications for amoxicillin recommendations. | Confounding factors, including the role of severe malaria in non-SAM deaths, are now more thoroughly discussed. We have also considered the implications of antibiotic resistance. | Discussion |
| 17 | Overlooks antibiotic stewardship concerns. | A section on antibiotic stewardship has been added, discussing concerns arising from widespread amoxicillin use. | Discussion |
| 18 | Recommendations lack specificity (e.g., how to optimize oxygen therapy). | Specific recommendations, including strategies for optimizing oxygen therapy, have now been added. | Discussion |
| 19 | Advocate for protocol revisions (e.g., timing of antibiotics, oxygen titration). | We now recommend protocol adjustments, including optimal timing for antibiotic administration and oxygen titration. | Discussion |
| 20 | Highlight need for resource allocation (e.g., amoxicillin access). | The revised manuscript emphasizes the need for adequate resource allocation, including improved access to essential drugs such as amoxicillin. | Discussion |
