# Supplementary material for: Comparison of time to death and its predictors among hospitalized children with and without severe acute malnutrition at Mulanje District Hospital, Southern Malawi
Source: PLoS One. 2025 Sep 30;20(9):e0333585. doi: 10.1371/journal.pone.0333585 (PMC12483230; doi:10.1371/journal.pone.0333585)
Supplement: Attachment — Submitted filename: The_response_to_reviewers(1)_auresp_5.docx [file pone.0333585.s007.docx]

**Point-by-point response file**

| **No.** | **Reviewer Comment** | **Response** | **Section** |
| --- | --- | --- | --- |
| 1 | If the reviewer comments include a recommendation to cite specific previously published works, please review and evaluate these publications to determine whether they are relevant and should be cited. There is no requirement to cite these works unless the editor has indicated otherwise. | All relevant publications have been thoroughly reviewed and critically evaluated. They are deemed appropriate for citation as they are directly relevant to the topic, methodologically sound, transparent, and provide valid evidence that supports or contrasts our findings. Their inclusion strengthens the scientific rigor and contextual background of the manuscript. | Whole manuscript |
| 2 | Please review your reference list to ensure that it is complete and correct. If you have cited papers that have been retracted, please include the rationale for doing so in the manuscript text, or remove these references and replace them with relevant current references. Any changes to the reference list should be mentioned in the rebuttal letter that accompanies your revised manuscript. If you need to cite a retracted article, indicate the article’s retracted status in the References list and also include a citation and full reference for the retraction notice. | All references have been carefully reviewed, updated, and verified for accuracy. They are now complete, current, and correctly formatted according to PLOS ONE guidelines. No retracted articles are cited.  Additionally, references numbered 46 to 52 were revised and updated to ensure correct citation and to accurately address comments from Dr. Dereje Tsegaye, hence enhancing the credibility and comprehensiveness of the reference list. | References |
| 3 | Your manuscript is almost ready for acceptance. However, we found one statement which we feel is unsupported: "This is aligned to the fact that males are biologically weaker than females, making them more susceptible to life risks, including death [28]". This is not supported by the cited study (nor would the reverse claim be supported by it). Consider removing this statement, or revising it to reflect better the findings of the referenced article. | The original citation [28] was incorrectly assigned. The correct reference is [27], which provides evidence supporting the statement that males are biologically more vulnerable than females.  The manuscript has been corrected accordingly, and the citation now accurately reflects the referenced study’s findings. This ensures the statement is fully supported by the literature. | Discussion and References |
